# A socio-ecological approach to reduce the physical activity drop-out ratio in primary care-based patients with type 2 diabetes: the SENWI study protocol for a randomized control trial

**DOI:** 10.1186/s13063-022-06742-7

**Published:** 2022-10-03

**Authors:** Guillem Jabardo-Camprubí, Judit Bort-Roig, Rafel Donat-Roca, Raimon Milà-Villarroel, Mercè Sitjà-Rabert, Jim McKenna, Anna Puig-Ribera

**Affiliations:** 1 Sports and Physical Activity Research Group, Centre for Health and Social Research, University of Vic-Central University of Catalonia, Sagrada Familia 7, Barcelona, VIC Spain; 2 School of Health Science UManresa, Sport Exercise and Human Movement (SEaMH), University of Vic-Central University of Catalonia, Av. Universitaria 4-6, Manresa, Spain; 3 School of Health Science Blanquerna, Ramon Llull University, Padilla, 326-332 Barcelona, Spain; 4 School of Health Science Blanquerna, Global Research on Wellbeing (GRoW) Research Group, Ramon Llull University, Padilla, 326-332 Barcelona, Spain; 5 School of Sport, Leeds Becket University, Leeds, LS1 3HE Leeds, England

**Keywords:** Physical activity, Health behaviour, Type 2 diabetes, Change of behaviour, Socio-ecological model; Nordic walking

## Abstract

**Background:**

Physical activity (PA) is a key behaviour for patients with type 2 diabetes (T2DM). However, healthcare professionals’ (HCP) recommendations (walking advice), which are short-term and individually focused, did not reduce the PA drop-out ratio in the long run. Using a socio-ecological model approach may contribute to reducing patient dropout and improving adherence to PA. The aim of this study is threefold: first, to evaluate the effectiveness of a theory-driven Nordic walking intervention using a socio-ecological approach with T2DM patients in Spain; second, to explore the feasibility on the PA adherence process in T2DM patients while participating in the SENWI programme; and third, to understand the HCPs’ opinion regarding its applicability within the Spanish healthcare system.

**Methods:**

A three-arm randomized control trial (*n* = 48 each group) will assess the efficacy of two primary care-based PA interventions (Nordic walking vs. Nordic walking plus socio-ecological approach; two sessions per week for twelve weeks) compared to a control group (usual HCPs’ walking advice on PA). Inclusion criteria will include physically inactive patients with T2DM, older than 40 years and without health contraindications to do PA. PA levels and drop-out ratio, quality of life and metabolic and health outcomes will be assessed at baseline, post-intervention and at 9- and 21-month follow-ups. The effect of the different interventions will be assessed by a two-factor analysis of variance: treatment group vs time. Also, a two-factor ANOVA test will be performed with linear mixed models for repeated measures.

A qualitative analysis using focus groups will explore the reasons for the (in)effectiveness of the new PA interventions. Qualitative outcomes will be assessed at post-intervention using thematic analysis.

**Discussion:**

Compared with the general PA walking advice and Nordic walking prescriptions, integrating a socio-ecological approach into Spanish primary care visits could be an effective way to reduce the PA drop-out ratio and increase PA levels in patients with T2DM. Such interventions are necessary to understand the role that multiple socio-complex process in day-to-day PA behaviour has in patients with T2DM in the Spanish context.

**Trial registration:**

ClinicalTrials.gov NCT05159089. Physical Activity Drop-out Ratio in Patients’ Living with Type 2 Diabetes. Prospectively registered on 15 December 2021.

**Supplementary Information:**

The online version contains supplementary material available at 10.1186/s13063-022-06742-7.

## Administrative information

**Table:** 

Title {1}	A socio-ecological approach to reduce the physical activity drop-out ratio in primary care-based patients with type 2 diabetes: The SENWI study protocol for a randomized control trial.
Trial registration {2a and 2b}.	NCT05159089. Physical Activity Drop-out Ratio in Patients’ Living with Type 2 Diabetes.
Protocol version {3}	Prospective registered on 15 December 2021. Last version registered on 13 June 2022.
Funding {4}	This protocol is funded by Col·legi de Fisioterapeutes de Catalunya.
Author details {5a}	Guillem Jabardo-Camprubí. Sports and Physical Activity Research Group, Centre for Health and Social Research, University of Vic-Central University of Catalonia, Sagrada Familia 7, Vic, Spain. Judit Bort-Roig. Sports and Physical Activity Research Group, Centre for Health and Social Research, University of Vic-Central University of Catalonia, Sagrada Família 7, Vic, Spain. Rafel Donat-Roca. School of Health Science UManresa. Sport Exercise and Human Movement (SEaMH). University of Vic-Central University of Catalonia, Av. Universitaria 4-6, Manresa, Spain. Raimon Milà-Villarroel. ﻿School of Health Science Blanquerna, Ramon Llull University, Padilla 326-332, Barcelona, Spain. Mercè Sitjà-Rabert. ﻿School of Health Science Blanquerna, Global Research on Wellbeing (GRoW) Research Group, Ramon Llull University, Padilla 326-332, Barcelona, Spain. Anna Puig-Ribera. Sports and Physical Activity Research Group, Centre for Health and Social Research, University of Vic-Central University of Catalonia, Sagrada Família 7, Vic, Spain.
Name and contact information for the trial sponsor {5b}	Col·legi de Fisioterapeutes de Catalunya; Segle XX, 78 (Barcelona) 08032 Spain. recerca@fisioterapeutes.cat
Role of sponsor {5c}	The role of the sponsor is to finance the protocol.

Trial registration data set from the World Health Organization

**Table:** 

**Data category**	**Information**
Primary registry and trial identifying number	ClinicalTrials.gov NCT05159089
Date of registration in primary registry	15 December 2021
Secondary identifying numbers	-
Source(s) of monetary or material support	Col·legi de Fisioterapeutes de Catalunya
Primary sponsor	Col·legi de Fisioterapeutes de Catalunya
Secondary sponsor(s)	n/a
Contact for public queries	GJC (guillem.jabardo@uvic.cat)
Contact for scientific queries	GJC (guillem.jabardo@uvic.cat)
Public title	
Scientific title	
Countries of recruitment	Spain
Health condition(s) or problem(s) studied	Type 2 diabetes mellitus, physical activity adherence
Interventions	Active comparator: Nordic Walking plus the socio-ecological model
	Active comparator: Nordic Walking intervention
	Control comparator: general nurses healthcare follow-up
Key inclusion and exclusion criteria	Ages eligible for study: ≥45 years Sexes eligible for study: both Accepts healthy volunteers: no
	Inclusion criteria: patients with type 2 diabetes
	Exclusion criteria: with contraindications to do physical activity
Study type	Interventional
	Allocation: randomized intervention model. Parallel assignment masking: single blind (evaluator)
	Primary purpose: treatment
	Phase I
Date of first enrolment	14-02-2022
Target sample size	144
Recruitment status	Recruiting
Primary outcome(s)	Physical activity drop-out ratio, physical activity levels, metabolic and health outcomes (HbA1c, blood pressure, waist circumference and BMI) and quality of life.
Key secondary outcomes	Socioeconomic status, social network index and demographic information (sex, gender, age, ethnic origin, marital and family status and medication)

## Background

Physical activity (PA)—understood as any body movement that requires energy expenditure above the resting metabolic rate—is a key behaviour for people living with type 2 diabetes mellitus (T2DM) [1, 2]. In these patients, increasing daily PA has a dose-response relationship with markers of glucose control (i.e., HbA1c) [3]. Moreover, being physically active also produces psychological and social benefits: it improves patients’ quality of life and reduces the healthcare costs associated with physical inactivity [4–6].

PA recommendations in patients with T2DM include at least 150–300 min of moderate-to-vigorous intensity (MVPA) most days of the week with a multicomponent training including aerobic and muscle-strengthening activities [7]. In addition, the amount of time spent being sedentary should be limited [8]. In this regard, adhering to regular PA—understood as not dropping out of the newly adopted PA behaviour [9]—is a way to control T2DM and promote self-management [10–12]. To achieve these goals, the preferred modality of health care professionals (HCP) to encourage and increase PA is walking. Walking 30 min a day is a popular recommendation for increasing PA and reducing time spent being sedentary that HCPs are able to make in health care consultations with these patients [13]. However, after decades of primary care-based PA recommendations and interventions, patients report dropout rates of 20–50% within the first 3–6 months of intervention [14, 15], and in the long run (e.g. 12–24 months), the interventions seem ineffective [16, 17].

To deal with this dropout problem, high-intensity interval training, which requires less time to achieve the same benefits as moderate intensity [18], has been proposed as a possible solution to overcome one of the most frequently reported barriers to adherence (namely, lack of time) [19, 20]. However, the advanced skills required and the necessary good physical condition to withstand these kind of protocols [21, 22] may not be a suitable solution for most patients [23]. Another option to reduce patients’ PA drop out is Nordic walking (NW) [24]. NW provides more health benefits (e.g. glucose control, cardio-respiratory fitness, flexibility and upper-body strength) than merely walking [25, 26], and it is easier to perform than high-intensity interval training [27]. However, although NW seems to be more enjoyable than simply walking, the PA drop-out ratio remains high [24].

Current interventions such as NW mainly focus on adopting PA recommendations without facing the main issue in public health: treatment adherence [28]. Gender, social support, built environment and sociodemographic context have been identified as relevant determinants for being physically active [29–31]. Studies reporting dropout from long-term PA programmes show that people with low education and socioeconomic status have a higher prevalence than their counterparts [14]. Moreover, a previous qualitative study identified that not only social inequity [32, 33] but also having non-negotiable kinship needs (e.g. family care) may effect treatment adherence [34].

In this context, HCPs’ poor results in making PA part of patients’ routine (i.e. adherence) may be due to the complexity of patients’ daily social life (e.g. structural barriers) where HCPs do not have any influence [9, 34, 35]. Structural barriers (e.g. political decision-making) may have more influence on PA routinization than patients’ motivation and knowledge [34, 36]. Yet, ineffective primary care-based PA recommendations may enhance socioeconomic barriers and health inequity in T2DM patients [32, 33].

PA adherence is a complex public health issue with multiple interacting influences. Thus, focusing on a single solution to improve PA adherence may be more helpful in achieving short-term and individual-level health outcomes than multiple long-term and population-level outcomes [37].

Using a socio-ecological model (SEM) approach in primary care-based PA practices may help to reduce patient drop-out rate and improve adherence to PA [38, 39]. SEM considers the complex interplay between individual, relationships, community and societal factors that influence both the adoption and the adherence of the treatment [40]. Thus, this approach focuses on changing the physical and social environments as well as modifying individual health-related factors [41]. This may involve facing different sociocultural barriers, including socioeconomic status and social inequity (e.g. delivering free guided intervention), gender- and age-specific needs (e.g. providing tailored information with a professional support), or built environment (e.g. enrolling neighbourhood green open spaces). However, HCPs might find it difficult to address such sociocultural and environmental barriers in short visits [11, 42]. Given the scale and span of the PA biological, psychological and social benefits for patients with T2DM, it is a public health priority to deploy more effective ways to implement day-to-day PA prescriptions in primary care practices.

This study protocol has two aims. The primary aim is to evaluate the effectiveness of a theory-driven Nordic Walking intervention—based on SEM (SENWI)—in people with T2DM that will be implemented in real-life primary care clinical practice in Spain. Second, we wish to explore the feasibility of the PA adherence process in T2DM patients while participating in the SENWI programme and to understand the HCPs’ opinion regarding its applicability within the Spanish healthcare system.

## Methods/design

### Study design

A mixed methodology will be used. First, a three-arm randomized control trial will compare the effectiveness of a 12-week SENWI programme, a NW programme and typical healthcare advice on PA. The main outcomes will be assessed at baseline, post-intervention and at 9- and 21-month follow-ups. Second, a qualitative thematic analysis using focus groups will aim to understand patient and HCP experiences of the SENWI intervention and factors that may enable or limit patients’ PA drop-out ratio, both of which will be assessed at post-intervention (T1). The study protocol (NCT05159089) was developed based on the Standard Protocol Items: Recommendation for Interventional Trials (SPIRIT) guidelines [43].

An intern Audit Committee will be made up of two research members (APR and RMV): a specialist in PA research and a statistician. This committee will verify the processes related to recruitment, informed consent, eligibility, assignment to the treatment groups and adherence to the intervention every month. The Committee will meet monthly and will support day-to-day organization of all research teams.

### Participants

A total of 144 participants (randomized in two intervention groups and one control group) will be included in this study. The study will be conducted in the Central Catalonia health area (Spain). Participants will be recruited from Monistrol de Montserrat *Primary Care Center* (CAP), Vic Sud CAP (Vic), CAP les Bases (Manresa), Hospital *Sant Bernabé* (Berga) and Anoia CAP (Igualada).

Inclusion criteria for eligibility will include participants (i) with T2DM, (ii) that have no major physical limitations prescribed by the doctor or any HCP and (iii) that are physically inactive according to the Spanish version of a Brief Physical Activity Assessment Tool – SBPAAT screening tool [44]. Participants will be excluded if they (i) are pregnant or have T2DM due to gestation; (ii) are unable to freely consent to take part in the study; (iii) are unable to understand the study materials or PA intervention; (iv) have complications such as neuropathy, retinopathy and nephropathy; (v) have relative or absolute contraindications to do PA; and (vi) have a body mass index over 34.9 kg/m^2^ since Nordic walking uses poles that require free arm mobility that is impaired by a body mass index over 35 kg/m^2^.

### Sample size

We calculated the sample size (*n* = 144) in relation to the drop-out ratio in PA interventions showed by other studies (15%) [45] with a balanced one-way analysis of variance power calculation of 80%, a significance level of 5%, an expected drop-out rate in participants of 15% and an effect size of 0.5. As a result, 48 participants will be needed in each group in the three-arm randomized controlled trial.

### Qualitative study sample size

A subsample for the qualitative study will be selected from those participants that will be enrolled in the intervention groups using a convenience sampling. Physiotherapists that are not part of the usual clinical team and that will deliver and prescribe PA during the intervention and HCPs already in contact with T2DM participants will also be engaged in the qualitative study. The collection of the sample will finish when saturation is achieved.

### Study procedure

Patients will be recruited by nurses at the CAPs, who will be informed personally at the CAP about the background and the aims of the study by the research team. Nurses who volunteer to participate will be given further information regarding eligibility criteria, and the researcher will give them a recruitment protocol to follow during the study.

Nurses from the CAPs were selected from potential trial participants during nurse’s consultations between February and April 2022. Then, in the recruitment site, the nurses will select participants for one of the three groups: (i) PA intervention using NW, (ii) PA intervention with SENWI and (iii) control group (general intervention given by HCP).

Patients that came to the consultations will be selected randomly, asking the odd number visits of the day to participate if they accomplish the inclusion/exclusion criteria. After the patients will be assessed as likely to benefit from PA interventions, an independent nurse that has not been previously in contact with the patient will explain the trial to interested individuals, give them an information sheet about the study and obtain their written informed consent approved by the ethics committee (in Catalan). The patients will be informed that participation in a PA (namely, Nordic walking) was required two times/week, highlighting that it would be good for them (physically, psychologically and socially). Those participants selected to the control group will be informed that they would be required to increase PA freely each day and that different outcomes would be registered over the following 24 months for the health and wellbeing of the patients. The informed consent will be obtained by the same nurses once the participant accepted to participate.

Participants in the quantitative study will be approached face-to-face for enrollment. When participants agree to participate, a face-to-face appointment with research members (GJC and JBR) will be scheduled. If any participant declines to participate, it will be registered and the reason for declining noted.

According to COREQ guidelines, and prior to formal data capture, the discussion-focus group process will be trialed three times to test and validate the discussion guide [46]. We will introduce open questions regarding participants’ beliefs to enrich the interview guide. In a spirit of continuous improvement, the guide will be reformulated and improved throughout the study after each meeting. Discussions will be terminated when the facilitator/interviewer feels that the research team could not develop more key themes.

Informed consent will be given voluntarily, and confidentiality of personal information followed the Protection of Personal Data and the guarantee of digital rights and the General Regulation (EU) 2016/679, of 27 April 2016, on data protection and complementary regulations. The Declaration of Helsinki statement of ethical principles for medical research involving human subjects will be followed [47].

### Patient public involvement

Patients had an involvement active role in the development of the protocol through a qualitative study carried out previously by the research team [34].

### Randomization and blinding

A computerized random-number generator (http:// www.randomization.com, created by Dr Gerard E. Dallal, Tufts University) will be used to randomize eligible participants at the level of the individual participant, stratified by sex and age, in blocks of ten.

Concealed randomization will be conducted by the research member after each participant has been included in the study, has been assigned an identification code and has completed the study baseline assessment. Nurses from CAPs/Hospitals will enrol the participants.

Allocation concealment will be ensured as the random-number generator will not be released until the patient has been recruited into the trial, which takes place after all baseline measurements have been completed. Participants will be notified personally face-to-face.

The design is open label with only outcome assessors being blinded so unblinding will not occur. The researchers carrying out the baseline assessments will be blind to group allocation. The statistician will also be blind to group allocation until the completion of the statistical analysis. Participants will be asked not to reveal group allocation when undergoing follow-up measurements. To assess the extent to which blinding has been preserved, researchers will record the number of cases in which allocation was revealed.

### Study interventions

**Fig. 1:**
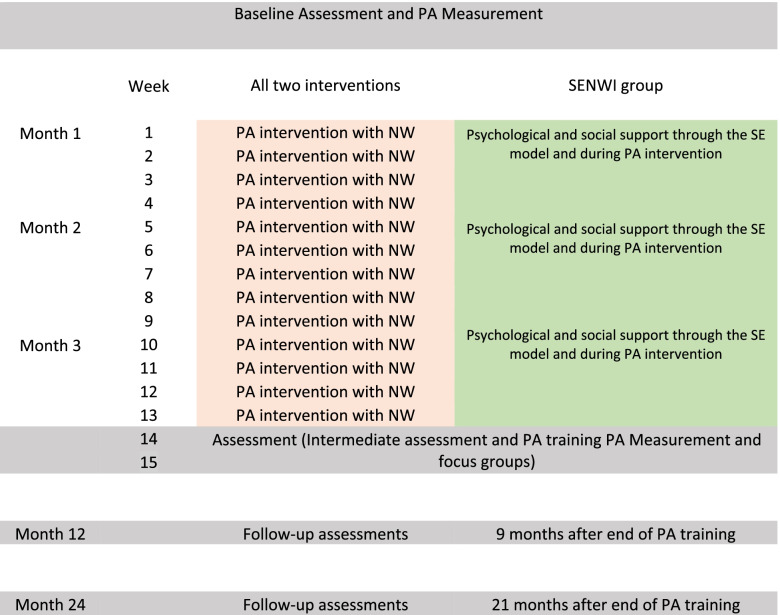
Schedule of the socio-ecological with Nordic walking intervention (SENWI) and Nordic walking intervention (NW). PA, physical activity

A three-arm randomized controlled trial will assess the impact of a primary care-based PA intervention implementing SENWI against an isolated NW programme (comparison group 1) (see Fig. 1) and usual PA advice (e.g. to walk) provided by HCPs (comparison group 2).

All instructors in charge of conducting the SENWI and NW interventions will undergo a standardized protocol. Both intervention (NW and SENWI) sessions will be implemented in the surroundings of the different CAP/hospital facilities (green areas or parks).

To assess safety and well-being during the period of the intervention, patients’ perceptions about health, injury, pain and intervention will be asked every week by a specially trained professional physiotherapist in charge of delivering the NW.

All participants will continue with their pharmacological treatment plan and nutritional and PA recommendations provided by the treating HCP. Moreover, all three interventions have no expected harmful effects as they only involve low- and moderate-intensity PA. However, it may be possible that some patients feel discomfort in the first weeks of the intervention. To deal with that, we propose a protocol that starts with less time and intensity and gradually increases. Also, instructors will be formed by HCPs (i.e. nurses) about hypoglycaemias and how to deal with it in case an event occurs.

Adverse events that the participant attributes as “study-related” will be collected at each follow-up assessment in both groups’ intervention. Healthcare use (number of visits to HCP) pertaining to the adverse event(s) will also be measured by research team members asking patients directly. To register healthcare use pertaining to adverse event(s), patients will be asked directly by research team members.

Adherence will be monetarised through attendance at the session schedule. Phone calls will be used to improve adherence to interventions.

### Comparator group 1: NW programme

Before the intervention, participants will do a beginner’s class in NW over 2 days with an instructor. This will be done at the same place where the intervention will be conducted (i.e. outdoors near the CAPs). After these two sessions, the 12-week intervention with two sessions/week will start. Sessions will be progressive: the first month will last for 30 min, the second month 35 min and the third month 40 min (plus 10-min warm-up and 10-min cool-down in each session). Track paths will be flat and will not have more than 100-m cumulative altitude. The meeting point will be at the CAP facilities.

Participants will be scheduled at two different times (e.g. midday and afternoon). Each group will receive 26 NW sessions. Patients will receive a schedule with all 12-week sessions.

### Comparator group 2: usual health care advice

The control group will receive the usual HCP’s advice on PA, health and illness management. This consists of checking up on the results of their blood analyses, asking questions about their day-to-day life, receiving HCP advice on how they should cope with hyperglycemia through diet and trying to encourage patients to walk and integrate more physical activities into their daily routines. Thus, HCP advice is grounded in general recommendations regarding PA and its importance, and not in how, when, where and with whom to do it.

### Intervention group: SENWI programme

Intervention group based on SEM will apply NW like the comparison group 1, but in this group instructors will add the SEM approach. The SEM approach is based on the *Caminemos!* (let’s walk!) protocol with adaptations to be applied in patients with T2DM [48, 49] (see Tables 1 and 2).

**Table 1:** General information about the study intervention

Name of the programme	Programme components	Person in charge of Training	Duration	General structure of each session
Comparator group 1	Increase PA levels throughout a NW protocol.	Physiotherapist instructor of NW.	12 weeks. Two sessions of NW per week of 30–40 min. 21-month post-intervention follow-up.	All sessions will begin with a 10-min warm-up focusing on joint mobility. The main session will last between 30 min (first month), 35 min (second month) and 40 min (third month). All training sessions will end with a 10-min cool-down using stretching of the lower limbs.
Intervention group	Raising awareness of the psychological and social importance of PA. Facing socioeconomic barriers to improve PA levels over time.	Physiotherapist instructor of NW trained from a SEM approach.	12 weeks. Two sessions of NW per week of 30–40 min. 21-month post-intervention follow-up.	All sessions will begin with a 10-min warm-up focusing on social and physical interactions and themes per week to work on (see Table L). The main session of NW will last between 30 min (first month), 35 min (second month) and 40 min (third month). All training sessions will end with a 10-min cool-down focusing on social interactions between participants, themes per week to work on and instructors answering questions.
Comparator group 2	Common approach used in healthcare.	HCP that is used to attending T2DM patients.	12 weeks and a 21-month follow-up	Common approach used in healthcare: recommendation to do more PA and walk more.

*PA* physical activity, *NW* Nordic walking, *SEM* socio-ecological model, *T2DM* type 2 diabetes mellitus

**Table 2:** Main themes to work on each week and curriculum objectives

Themes per week	Objectives
1–2	Introduce the idea that being physically active should be part of normal ageing and should continue at any age and in any health condition (e.g. T2DM). Teach that ageing or T2DM itself does not cause decreased PA. Identify barriers to being more physically active. Differentiate between modifiable (e.g. psychological) and unmodifiable (e.g. sociological) causes.
3–4	Reinforce the idea that being physically active should be part of everyday normal life and should continue under any circumstances. Identify common changes associated with T2DM and teach that with modifications one can resume an active life.
5–6	Teach that being unable to learn a new habit is not caused by T2DM or ageing. Introduce the idea of SEM. Reflect on whether expectations and beliefs about T2DM have changed. Enhance social networks that support PA
7–8	Reinforce key concepts. Identify positive aspects about being physically active and negative aspects about not being physical active. Problem-solve on how to maintain a PA over time. Enhance social networks that support PA.
9–10	Reinforce the idea that being physically active should be part of everyday normal life and should continue under any circumstances. Identify barriers to being more physically active. Enhance social networks that support PA
11–12	Reinforce key concepts. Problem-solve on how to maintain a PA over time. Make individual commitments to increase PA taking into consideration psychological and social barriers. Enhance social networks that support PA

*PA* physical activity, *T2DM* type 2 diabetes mellitus, *SEM* socio-ecological model. Adaptation from *Caminemos!* (Let’s walk!) protocol [46]

To deliver this model, instructors will be formed by the principal investigator. In each session, the instructors will give small talks and lead a group discussion about the key themes before, during and after the SENWI programme. Themes are divided into three general blocs: enhance the need to do PA continually throughout life, problem-solve on how to maintain PA over time and enhance sociability and social networks that support PA. Moreover, during the first two weeks participants will be asked to report the reasons for being insufficiently active in order to enhance the need to do PA, and then they will be taught to categorize the reasons as either modifiable (e.g. not having a partner to exercise with) or nonmodifiable (e.g. being old or having a medical condition). Then, participants will be asked to find solutions to different problems together as a group, and to explain how to address the modifiable reasons for being insufficiently active. Finally, in each session participants will be asked to establish action plans to increase PA and made commitments to do a specific action before the next meeting (e.g. take my grandson to school on foot). All participants will be encouraged to write down how much “exercise” they do each day and comment at the beginning of each session on how well they kept their commitment. They will also be asked to record any barriers and enablers that they encountered, as well as the extent to which they were able to overcome these barriers.

### Quantitative and qualitative outcomes

Quantitative assessments will be conducted by researchers at the following time points: T0 = baseline preintervention, T1 = at month three when the intervention has finished (post-intervention), T2 = at month twelve (9-month follow-up) and T3= at month 24 (21-month follow-up) (Table 3 shows the SPIRIT table). All researchers in charge of conducting the assessments will take a standardized training session.

**Table 3:**
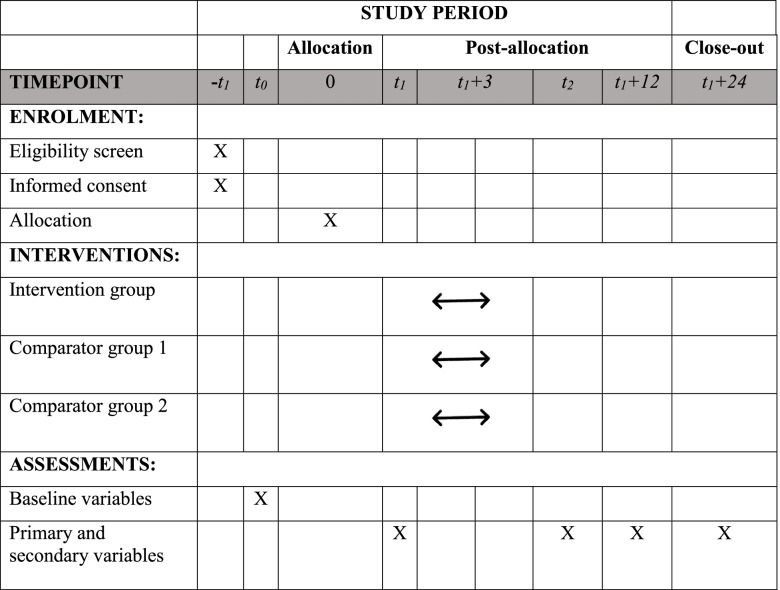
Study timeline

Primary outcomes include (i) PA drop-out ratio; (ii) PA levels; (iii) metabolic and health outcomes (HbA1c, blood pressure, waist circumference and BMI); and (iv) quality of life (SF-12) (see Table 4).

**Table 4:** Overview of outcomes, outcome measures, instruments and assessment time points

Outcomes	Outcome measures	Instrument/who measures	Assessment time point
Personal information	Age, gender, civil status, living arrangement, educational background, medical conditions, smoking and alcohol habits.	Primary care records/self-report	T0
Primary clinical trial outcomes			
●Physical activity	Daily counts per minute and intensity of exercise, and daily step counts.	ActivPal®/research team	T0, T1, T2, T3
●Physical activity drop-out ratio	Less of 80% of attendance during the intervention or auto-reported drop out after intervention (yes/no)	Excel/research team	T0, T1, T2, T3
●Quality of life	Physical function, physic role, corporal pain, general health, vitality, social function, emotional role and mental health	SF-12 questionnaire/research team	T0, T1, T2, T3
●Blood pressure	Systolic and diastolic blood pressure; heart rate	OMRON M6 comfort/nurse	T0, T1, T2, T3
●Blood glucose levels (last 3 months)	HbA1c	CATSALUT Dataset	T0, T1, T2, T3
●Anthropometry	Waist circumference and BMI	Measuring tape and coefficient between weight and height/nurse	T0, T1, T2, T3
Secondary clinical trial outcomes			
●Socioeconomic status	Job, education and income	Job and education level and individual and family annual income/research team	T0
●Social network index	Number of social interactions with family friends or mates	Berkman et al. [47] questionnaire with adaptations [48]/research team	T0
●Demographic and background information	Sex, gender, age, ethnic origin, marital and family status or obligations and medication	Self-report information/nurse	T0, T1, T2, T3 (only apply times for medication)
Primary qualitative study outcomes			
●HCP and T2DM perspective on the intervention	Perceptions and experience to examine the feasibility about the intervention and applicability to everyday life	Focus groups /research team	T1

Assessment time points: T0 = baseline pre-intervention, T1 = at month three of intervention, T2 = at month 12 (9 months after the end of the intervention), T3 = at month 24 (21 months after the end of the intervention). *HCP*, health care professionals; *T2DM*, type 2 diabetes mellitus

Demographic outcomes include (v) socioeconomic status and social network index and (vi) demographic and background information that will include sex, gender, age, ethnic origin, marital and family status or obligations and medication (see Table 4).

### PA drop-out ratio

During the intervention, dropout will be considered as less than 85% attendance of scheduled sessions [50].

After the intervention, PA dropout will be measured by asking the participants if they continue to undertake any kind of PA in their daily life. The answer will be registered as yes or no, and it will be noted what kind of PA is done. Participants will be asked once a month until the 21-month post-intervention follow-up through telephone calls and messages. In addition, the SBPAAT screening tool will be used to register whether participants are active or inactive at the follow-ups (*T* = 1, 2 and 3).

### PA assessment

MVPA, light intensity PA (LIPA), standing, total sitting time and sitting bouts will be assessed by the activPAL3™ micro (PAL Technologies Ltd., Glasgow, UK), weighing 9g and measuring 25 × 45 × 5mm [51, 52]. The activPAL^TM^ will be placed in a small flexible nitrile sleeve to waterproof the device and will be attached to the participants’ right thigh using a transparent film (10 × 10cm of hypoallergenic Tegaderm^TM^ Foam Adhesive Dressing). Participants will be instructed to wear the device for 24 h per day during a 7-day period. The recording time will begin at 12am for all participants. Participants will be asked to go to the CAP facilities to fit the activePAL^TM^. A research team member will fit the activPAL^TM^ on the participants’ thighs and will provide them with two additional dressings in case reattachment is required (instructions on how to change the dressings will be explained). Participants will have a field diary to note down if there is any problem with the activePAL^TM^, and to note the time they go to bed and wake up.

The protocol that will be used for data collection and analysis is described in detail elsewhere [53]. Briefly, data will be included in the analyses of the participants who provide a minimum of 4 valid days of recording (including one weekend day) [54]. Valid days will be defined as a day with ≤4h of non-wear time during identified walking hours. Non-wear time is defined as a period with ≥60min of consecutive zero activity counts. Data will be analysed using activPAL^TM^ Professional Software^TM^ (version 7.2.32) and further processes using Microsoft Excel 2010 (Redmond, WA, USA) and METLAB (MathWorks®, Natick, MA, USA).

### Metabolic and health outcomes assessment

Patients will be asked to consent to their register of the HbA1c results, and the indications of medication treatment for cholesterol, high blood pressure, or T2DM (e.g. metformin), before, during and after the intervention of the health system (CATSALUT dataset). Blood pressure will be measured manually using a stethoscope and a blood pressure cuff (Omron Monitor Ref.171994.2 M2). The subject will have to sit still for 5 min before blood pressure measurement.

Waist circumference will be measured between the iliac crest and the lowest rib in patients’ standing position using a flexible measuring tape (Perimeter tape SECA Ref: 039-SA201.171.7009). BMI will be calculated as weight in kilogrammes divided by height in squared metres (kg m^−2^)(Romana SECA 700 C/Tallimetro weighing machine). The same experienced nurse will take the blood pressure and anthropometric measurement at the beginning of the study (*T* = 0) and at the follow-up measures (*T* = 1, 2 and 3) to avoid different individual measuring techniques [55].

### Quality of life

Quality of life will be measured using the SF-12 questionnaire (Spanish version) [56]. This questionnaire assesses the quality of life related to health and has 12 items that assess eight dimensions: physical function, physical role, corporal pain, general health, vitality, social function, emotional role and mental health. The SF-12 will be administered by nurses and questions related to it will be answered. Answers follow a Likert scale format and the final score ranges between 0 and 100, where a higher score means a higher quality of life related to health. Patients will complete the SF-12 questionnaire before and after the intervention, and 21 months after intervention.

### Socioeconomic status and social network index

Socioeconomic status will be assessed by occupation, education level and annual income [54]. Occupation will be assessed as a categoric variable (job, no job, retired); education level will be assessed by taking into consideration the highest educational qualification (e.g. degree); and annual income will be assessed [54].

The social network index will be assessed using the adapted Berkman et al. questionnaire [57] on the number of interactions with family, friends, workmates, neighbourhood groups or social groups (e.g. sport clubs).

Social network and socioeconomic status will be recorded by nurses at the beginning of the study during medical consultations and after the intervention at 24 months (21 months after the intervention).

### Patients’ intake of medication

Participants will be asked to agree to use their clinical history to register this outcome in the written informed consent. The same nurses in charge of the caption and randomization of participants will be in charge of recording patients’ intake of medication.

### Patient and HCP experience

Focus groups will be conducted by the research team at T1. The qualitative study will explore the following: (i) HCP opinion regarding the applicability of SENWI in the Spanish healthcare system and (ii) T2DM patients’ perceptions and experience to examine the feasibility of PA adherence in the SENWI protocol. We will include (i) HCPs that participate in the study to deliver the SENWI protocol, (ii) patients with T2DM in the SENWI group that finish the protocol and (iii) patients with T2DM in the SENWI group that did not finish the protocol.

## Data collection and management

All assessments and collection of outcomes will be conducted at the CAP facilities. Nurses will collect all data except activePAL^TM^ and qualitative data. Qualitative data will be collected by the research members (RCP and PFF) at the end of the intervention (*T* = 1). Focus groups for qualitative data will be delivered face-to-face by two research staff members (GJC to undertake the focus group and JBR to take notes), tape-recorded and transcribed verbatim. Overall data management will be done by the research members’ staff at the University of Vic-Central University of Catalonia.

The number of participants that decline to participate in the study and those withdrawing during the course of the study will be tabulated by reason for not participating, and by reason for withdrawing (voluntarily given). Confidentiality of personal information about potential and enrolled participants will be protected using codification (number of participants; number of groups; number of outcome assessments) before, during and after the trial by the principal investigator (GJC) at a University of Vic-University of Central Catalonia computer server.

### Data analysis

Descriptive statistics will be reported as the mean and standard deviation (SD) for quantitative variables (PA and metabolic and health assessment outcomes) and using frequency rates and percentages for qualitative variables (drop-out ratio, medication, quality of life, socioeconomic status and social network). The Shapiro–Wilk test will be used to assess the normality of quantitative variables. For each outcome variable considered, the effect of the three different interventions will be assessed by a two-factor analysis of variance: the first factor will be the treatment group and the second factor will be time. To determine whether one treatment improves the scores of the study variables compared with the other, two-factor ANOVA tests will be performed with linear mixed models for repeated measures. An odds ratio and hazard ratio will be performed comparing drop outs in SENWI, NW and the control group at 3, 9 and 21 months.

Socioeconomic status and social network index will be analysed and compared in relation to the PA attendance at 3, 9 and 21 months post-intervention using the non-parametric chi-square test (*χ*^2^) and logistic regression when appropriate.

Demographic and other patient characteristics recorded (e.g. age, gender, marital status, income, education and employment) will be tabulated and analysed as covariables alongside the PA levels and PA drop-out ratio at the beginning and at 21 months post-intervention. Correlations and differences between groups and these variables will be analysed. Difference in baseline levels of outcome variables (PA, drop out, HbA1c and so forth) will be identified, and the correlation with the different covariables will be assessed.

A *p*-value of < 0.05 will be considered statistically significant. When multiple comparisons are carried out, the Bonferroni correction will be applied. All analyses will be carried out with the obtained data without employing missing data replacement techniques. Analysis will follow intention-to-treat principles. Assumptions will be tested regarding missing data, randomization and contamination. All analyses will be carried out using the R version 3.6.1 software.

The individual intervention will be regarded as successful if (i) it leads to an average increase in activity and (ii) the participant did not drop out of the PA (NW or any kind of PA) in the long run (i.e. if the patients answer yes when asked if they still do PA of some kind).

The SENWI programme will be regarded as improving usual healthcare if it increases PA at any level [2]. We will assess changes between groups in indicators relating to people living with T2DM health issues including HbA1c, weight, waist circumference and blood pressure. Finally, comparison between groups variables at 9 and 21 months will be needed to see whether the SENWI protocol is effective or not, and to determine if the PA drop-out ratio increases after removing the social and economic support (i.e. study intervention), in relation to the NW and SENWI.

To establish how the intervention works and for whom moderator and mediator analysis will be conducted. Suspected moderators will include participants’ demographic factors (e.g. socioeconomic status) and baseline intentions to engage in activity (e.g. sociocultural needs).

### Qualitative data analysis

Qualitative analysis will be performed with the Atlas.ti software. Thematic analysis will be used to develop themes and categories in the qualitative database in social theory [58, 59]. Two researchers with experience in qualitative research will perform analyses independently and then discuss and agree on key themes (JBR and GJC). A six-phase method will be as follows [60]: (1) two researchers will independently code the same subset of transcripts (10%), familiarizing themselves with the data, and (2) generate the initial codes; (3) members of the research team will discuss interim themes and reach a consensus; (4) similar coding schemes will be used for the qualitative data collected via focus groups, but where one set of results uncovers a theme not covered by other results, additions may be made; (5) a priori categories and themes developed from the data will be defined and named; and finally, (6) results and interpretations will be discussed by research members and the report will be written.

Transcripts and themes will be returned to all research members’ staff for verification [61]. Patient and HCP quotes to support the results will be identified and translated from Catalan/Spanish to English.

Credibility of the data will be achieved by reviewing the documents handwritten by the participants and using their complementary comments, as well as the prolonged commitment of the researcher with the data. In addition, two experts in charge of research quality will supervise and audit the entire research process. By using a combination method, that is of interviews and field notes, as well as sampling with the maximum variation, data transferability will be achieved [62].

## Discussion

This study proposes a comprehensive approach, taking into consideration multiple levels of influence on PA adherence, to find out solutions for HCPs to prescribe PA efficiently in patients living with T2DM. First, we want to evaluate the effectiveness of a theory-driven PA intervention based on SENWI in people living with T2DM that will be implemented in real-life Spanish primary care clinical practice. Second, we wish to explore the feasibility on the PA adherence process in T2DM patients while participating in the SENWI programme, and to understand the HCPs’ opinion regarding its applicability in the Spanish healthcare system. As a result, the applicability of this study will be the preparation of easy and friendly guidelines for HCPs to apply SENWI during consultations.

PA interventions for patients with T2DM have been focused on the type of training (e.g. walking) [24] or intensity (e.g. high-intensity PA) [23, 63]. Despite their effectiveness in adopting a new behaviour, PA adherence has not yet been addressed. As these interventions ignore the social complexity of PA, poor results have been gathered in the last decade of research to be used in day-to-day life in patients with T2DM [64, 65]. More than 40% of patients do not adhere in the long run to HCP recommendations on lifestyle changes when they include PA [28]. As a result, although PA is a key behaviour for patients with T2DM to manage their illness, HCPs feel powerless to prescribe PA efficiently to improve adherence and avoid dropout [42].

Our hypothesis is that the SENWI approach may be useful to HCPs to deal more efficiently with PA adherence in patients with T2DM in social complex situations. For example, instead of just motivating or informing patients about the importance of PA, HCPs should recommend how to avoid barriers related to PA (e.g. helping to re-schedule sessions in case some problem arises or proposing other PA activities to do in their daily lives), where to do it (e.g. open green areas or parks) and with whom (e.g. group activities). Therefore, we propose that HCPs should consider the different levels of influence that could affect T2DM patients’ PA adherence. To do that, other studies have proposed a three-step ‘action management’ approach: prepare the user, structure the action and design the context [66].

Using this ‘action management’ approach, ‘prepare the user’ might involve encouraging those who intend to exercise to wear the right footwear as daywear thus allowing for PA at any time, while also learning how to self-manage confidence, support persistence and solve problems in daily life. ‘Structure the action’ could involve doing activities where their demands are known and within their capacity (e.g. flat walking routes) and knowing how to make PA easier if it should become overly demanding. Instead of responding to changing contexts (i.e. non-modifiable contexts) by doing no PA, ‘design the context’ could involve exercising with others and increasing the sociability by talking about and discussing factors that hinder and facilitate PA [67, 68]. HCPs also need to become skilled in using, and knowing how to introduce, these same issues into their consultations to optimize their value to people living with T2DM. Otherwise, the responsibility to deal with PA adherence relies on both powerless patients living with T2DM and HCPs [11, 69].

The SENWI programme will employ these three elements to help T2DM patients to adopt and maintain PA in their daily routines and cope with perceived barriers and enablers. Being more physically active may improve patients’ health: increasing daily PA has a dose-response relationship with markers of glucose control (i.e. HbA1c) [3], produces psychological and social benefits and also improves patients’ quality of life and reduces the healthcare costs associated with physical inactivity [4–6].

Moreover, it may help HCPs, who are a key influence on patients’ health behaviours [11], and it could become a protocol to be used in the healthcare Spanish system that may reduce, in the long run, the total healthcare cost to manage and control this pathology (i.e.T2DM) [6, 70].

### Strengths and limitations

To our knowledge, this is the first study that implements a SEM approach to incorporate PA in patients living with T2DM during Spanish primary care visits. Moreover, it focuses on HCPs to help them in their day-to-day consultations to prescribe PA. We wish to know not only its effectiveness during the intervention (12 weeks), but also its effectiveness in real-life situations once the interventions have finished (at 9 and 21 months post-intervention).

The prospective collection of data with a 21-month follow-up outcome is one of the strengths of this study. The short intervention with the participants to deal with drop-out and adherence (only 3 months) and the possibility of the heterogeneity of the participants (e.g. not-working and working participants) are two of the limitations. However, to deal with these limitations, the second strength is that we propose to use a qualitative perspective within the clinical trial in clinical practice reality [71, 72]. We propose a mixed methods approach to understand the quantitative data with a qualitative perspective to understand the experiences of both patients and HCPs and how this programme can be integrated into the Spanish healthcare system from the users and HCP perspective.

## Conclusions

Compared with the general PA walking advice and Nordic walking prescriptions, integrating a SEM approach into Spanish primary care visits could be an effective way to reduce the PA drop-out ratio and increase PA levels in patients with T2DM. Such interventions are necessary to understand the role that multiple socio-complex process in day-to-day PA behaviour plays in patients with T2DM in the Spanish context.

## Trial status

The call for participants in Monistrol de Montserrat CAP began on 1 February 2022. We had enrolled 40 volunteers at the time of submission of the manuscript. Recruitment is expected to start at Hospital Sant Bernabé in September 2022, at Vic Sud CAP in November 2022, at Anoia CAP in January 2023 and at CAP les Bases in February 2023, at Anoia CAP. Data analysis will be completed by December 2023. Trial status: Recruiting participants.

## Ancillary and post-trial care

There is no anticipated harm and compensation for trial participation. However, participants will be able to contact the research team at any moment after the intervention as post-trial care.

## Supplementary Information

**Additional file 1.** Ethical committee approval.

**Additional file 2.** Funding documentation.

**Additional file 3.** SPIRIT checklist.

**Additional file 4.** Inform consent materials (in Catalan).

**Additional file 5.** Inform consent materials (in English).

## Data Availability

All research team members will have access to the trial dataset. Full protocol may be available upon request.

## Abbreviations

T2DM: Type 2 diabetes mellitus
PA: Physical activity
CAP: Primary Attention Centre
HCP: Healthcare professionals
SEM: Socio-ecological model
SENWI: Socioecological Nordic Walking Intervention
HbA1c: Glycate haemoglobin
SD: Standard derivation
NW: Nordic walking
BMI: Body mass index
