# Supplementary material for: A socio-ecological approach to reduce the physical activity drop-out ratio in primary care-based patients with type 2 diabetes: the SENWI study protocol for a randomized control trial
Source: Trials. 2022 Oct 3;23:842. doi: 10.1186/s13063-022-06742-7 (PMC9531392; doi:10.1186/s13063-022-06742-7)
Supplement: Supplementary file 1 — Additional file 1. Ethical committee approval. [file 13063_2022_6742_MOESM1_ESM.pdf]

CER
Comitè d'Ètica de la Recerca
UVIC·UCC

Favourable report
Research project
Internal code: 177/2021
13 June 2022

Dr Ester Busquets Alibés
Technical Secretary of the UVic-UCC Research Ethics Committee

CERTIFIES

That at its meeting of 4 October 2021, the UVic-UCC Research Ethics Committee considered the research project:

Title: A socio-ecological approach to reduce the physical activity drop-out ratio in primary care-based patients with type 2 diabetes: The SENWI study protocol for a randomized control trial

Internal code: 177/2021.
Lead researcher: Guillem Jabardo Camprubí.

Considers that:

– It meets suitability requirements in relation to objectives and design methodology.
– It meets ethical requirements for obtaining informed consent and aspects relating to confidentiality.
– The competence of the lead researcher and the resources available are appropriate to carry out the study.

Consequently, this Research Ethics Committee has issued a FAVOURABLE REPORT[1].

Ester Busquets Alibés - DNI 33946176Q (TCAT)

Firmado digitalmente por Ester Busquets Alibés - DNI 33946176Q (TCAT)
Fecha: 2022.06.13 13:26:36 +02'00'

13/06/2022

Signed by the Technical Secretary

[1]A favourable report places the following obligations on the lead researcher:

a) If necessary, to submit the project to external competitive or non-competitive calls with the same key features that have been favourably considered by this Research Ethics Committee.
b) If necessary, to carry out the project with the same key features that have been favourably considered by this Research Ethics Committee.
