# Supplementary material for: A socio-ecological approach to reduce the physical activity drop-out ratio in primary care-based patients with type 2 diabetes: the SENWI study protocol for a randomized control trial
Source: Trials. 2022 Oct 3;23:842. doi: 10.1186/s13063-022-06742-7 (PMC9531392; doi:10.1186/s13063-022-06742-7)
Supplement: Supplementary file 2 — Additional file 2. Funding documentation. [file 13063_2022_6742_MOESM2_ESM.pdf]

Col·legi de Fisioterapeutes de Catalunya

Daniel Jiménez Hernandez, Secretary of the Board of Government of the Col·legi de Fisioterapeutes de Catalunya, with Head Office at 78 Segle XX street, Barcelona, 08032.

CERTIFIES

That Mr. Guillem Jabardo Camprubí with collegian number 9613 applied for a grant, as principal researcher, at the Call for Research Fund 2021 of the Col·legi de Fisioterapeutes de Catalunya for the project, "***Effectiveness in promoting and prescribing exercise in patients with type 2 diabetes: a multicomponent intervention based on the model of basic social processes through Nordic Walking***" which included the protocol: "***A Socio-ecological approach to reduce physical activity drop-out ratio in primary-care based patients with type 2 diabetes: The SENWI study protocol for a randomized control trial***".

This project with register number R05/21 was awarded with a research grant of 5990,75€ on December 13th, 2021.

For the record, this certificate is issued and sent on request of the interested person.

Barcelona, June 17th, 2022

Firmado digitalmente por Daniel Jiménez
Nombre de reconocimiento (DN): cn=Daniel Jiménez, o=JG, ou=Secretari, email=secretari@fisioterapeutes.cat, c=ES
Fecha: 2022.06.17 15:59:49 +02'00'
