# Supplementary material for: A socio-ecological approach to reduce the physical activity drop-out ratio in primary care-based patients with type 2 diabetes: the SENWI study protocol for a randomized control trial
Source: Trials. 2022 Oct 3;23:842. doi: 10.1186/s13063-022-06742-7 (PMC9531392; doi:10.1186/s13063-022-06742-7)
Supplement: Supplementary file 4 — Additional file 4. Inform consent materials (in Catalan). [file 13063_2022_6742_MOESM4_ESM.pdf]

Informació als participants

Els membres de l'equip d'investigació de la *Universitat de Vic-Universitat Central* de Catalunya, dirigits per l'investigador principal *Guillem Jabardo Camprubí*, estem portant a terme un projecte d'investigació: **Efectivitat per a prescriure exercici físic en pacients amb diabetis tipus 2: una intervenció multicomponent basada en el model dels processos socials bàsics a través de la Marxa Nòrdica.**

El projecte vol investigar <u>beneficis de l'activitat física en la salut en pacients amb diabetis tipus 2.</u> Per a realitzar-ho s'ha de portar a terme un assaig clínic en el qual es demanarà als participants poder accedir a les seves dades de salut i alhora a participar en activitats de marxa nòrdica dos cops per setmana durant un període de tres mesos.

En el projecte participen investigadors els següents centres d'investigació: Universitat de Vic-Universitat Central de Catalunya i del CAP de Sant Joan de Barcelona. En el context d'aquesta investigació li demanem la seva col·laboració per participar en aquest estudi de marxa nòrdica i salut ja que vostè compleix els següents criteris d'inclusió:

- Persones de 40 a 65 anys (època laboral dels pacients: entre el moment on pot començar a aparèixer la patologia al moment de la jubilació)
- Amb diagnòstic de DT2 establert amb més de 2 anys des del diagnòstic
- Signatura del consentiment informat

Aquesta col·laboració implica participar en dos sessions de marxa nòrdica a la setmana durant tres mesos i en la presa de variables un total de tres cops: abans de la intervenció, just després i al cap de dotze mesos.

Tots els participants tindran assignat un codi amb el qual serà impossible identificar al participant amb les respostes o dades obtingudes, germanitzant la confidencialitat en tot moment. Les dades que s'obtinguin a través de la participació no s'utilitzaran amb altres finalitats diferents a les explicades en aquesta investigació i passaran a formar part d'un fitxer de dades del que serà màxim responsable l'investigador principal. Aquestes dades protegides mitjançant la codificació en un document Excel encriptat amb contrasenya que serà únicament visible per l'investigador principal.

El fitxer de dades de l'estudi estarà sota la responsabilitat de l'investigador principal, davant del qual es podrà exercir en tot moment els drets que estableix la Llei Organica 3/2018, del 5 de desembre, de la Protecció de Dades Personals i garantia dels drets digitals i el Reglament general (UE) 2016/679, del 27 d'abril de 2016, de protecció de dades i normativa complementaria.

Ens posem a la seva disposició per resoldre qualsevol dubte que pugui sorgir. Pot contactar amb nosaltres a través del correu electrònic de l'investigador principal: guillem.jabardo@uvic.cat

CONSENTIMENT INFORMAT

Jo, ____ , major d'edat, amb DNI ________________________ actuant en nom i interès propi

Declaro que:
He rebut informació del projecte (títol) del que se m'ha entregat la fulla informativa annexa a aquest consentiment i pel qual es sol·licita la meva participació. He entès el que significa, se m'han resolt els dubtes i se m'han explicat les accions que es derivaran del projecte. Se m'ha informat de tots els aspectes relacionats amb la confidencialitat i protecció de dades en compliment de la Llei Orgànica 3/2018, del 5 de desembre, de Protecció de Dades Personals i garantia dels drets digitals i el Reglament general (UE) 2016/679 del 27 d'abril de 2016 de protecció de dades i normativa complementaria.

La meva col·laboració en el projecte es totalment voluntària i tinc dret a retirar-me en qualsevol moment, revocant aquest consentiment, sense que això pugui influir negativament en la meva persona en cap cas. En cas de revocar el consentiment, tinc dret a que les meves dades siguin esborrades de l'arxiu d'estudi.
Així mateix, renuncio a qualsevol benefici econòmic, acadèmic o de qualsevol altre naturalesa que es podria derivar del projecte i els seus resultats.

Per tot això,
DONO EL MEU CONSENTIMENT A: ____________________________________
Participar en el projecte: **Efectivitat per a prescriure exercici físic en pacients amb diabetis tipus 2: una intervenció multicomponent basada en el model dels processos socials bàsics a través de la Marxa Nòrdica.**

Que l'equip d'investigador de la Universitat de Vic-Universitat Central de Catalunya i l'investigador principal Guillem Jabardo Camprubí puguin gestionar les meves dades personals i difondre la informació que el projecte generi. Es garantissin que es preservarà en tot moment la meva identitat i intimitat, amb les garanties establertes per la Llei Orgànica 3/2018, del 5 de desembre, de Protecció de Dades Personals i garantia dels drets digitals i el Reglament general (UE) 2016/679 del 27 d'abril de 2016 de protecció de dades i normativa complementaria.

Que l'equip d'investigadors dirigits per Guillem Jabardo Camprubí conservarà tots els registres efectuats sobre la meva persona en un suport digital-electrònic, amb les garanties i els terminis legalment previstos, si estan establerts, i a falta de previsió legal, durant el temps que fos necessari per complir les funcions del projecte per el qual les dades van ser obtingudes.

A ___________________________ *a* ____ *de*______________ *de*______

Firma participant: *Firma IP:*
