# Supplementary material for: A socio-ecological approach to reduce the physical activity drop-out ratio in primary care-based patients with type 2 diabetes: the SENWI study protocol for a randomized control trial
Source: Trials. 2022 Oct 3;23:842. doi: 10.1186/s13063-022-06742-7 (PMC9531392; doi:10.1186/s13063-022-06742-7)
Supplement: Supplementary file 5 — Additional file 5. Inform consent materials (in English). [file 13063_2022_6742_MOESM5_ESM.docx]

**Participants information**

The research team members of the *University of Vic – Central University of Catalonia,* led by the principal investigator *Guillem Jabardo Camprubí*, are conducting a research project named: ***Effectiveness in promoting and prescribing exercise in patients with type 2 diabetes: a multicomponent intervention based on the model of basic social processes through Nordic Walking.***

The research project aims to comprehend the physical activity benefits in health in patients that live with type 2 diabetes mellitus. To do this, a clinical trial must be carried out. Participants will be asked: i) to consent research team to look at their health data; ii) to wear an accelerometer pre-intervention, post intervention and one year and two years post intervention; and iii) to participate in one of the study groups (control group, Nordic walking with SENWI approach or Nordic Walking). If participant is allocated at one of both Nordic walking group, they will have to undertake this activity twice a week for a period of three months.

In the context of this research, we ask for your collaboration to join it if the following criteria are accomplished:

- Having more than 40 years old and without any contradiction to do physical activity (assessed by the doctor).
- More than 2 years of a type 2 diabetes since diagnosis
- Signed inform consent

All participants will have a code which will facilitate all data confidentiality. All the data from this study will not be used for other propose that have not been specify by researchers. All data will be stored with the principal investigator at the University of Vic – Central University of Catalonia in an encrypted computer folder.

All responsibility of the study data will be under the principal investigator, respecting all time the Law: (Llei Organica 3/2018) from 5^th^ of December, of Protection Personal Data and will guaranty de digital rights of the General Regalement (UE) 2016/679, from 27^th^ of April from 2016.

We are at your disposal to resolve any doubts that may arise. You can contact us through the principal investigator’s e-mail: guillem.jabardo@uvic.cat

**INFORMED CONSENT**

I, _____________________________________________________, of age, with ID identification ___________________________________________ with freedom of choice and in my interest

I declare that:

I have received information about the project title ***Effectiveness in promoting and prescribing exercise in patients with type 2 diabetes: a multicomponent intervention based on the model of basic social processes through Nordic Walking*** of which I have been given the information sheet attached to this consent and for which my participation is requested. I understand what it means, my doubts have been resolved and the actions that will derive from the project have been explained to me. I have informed of all aspects related to confidentiality and data protection in compliance with Organic Law 3/2018, of December 5, on the Protection of Personal Data and guarantee of digital rights and General Regulation (EU) 2016/679 of April 27, 2016 data protection and complementary regulations.

My collaboration in the project is completely voluntary and I have the right to withdraw in

at any time, revoking this consent, without this having a negative influence in my person in any case. In the event of revoking consent, I have the right to my data are deleted from the study file. Likewise, I renounce any financial, academic or any other kind of benefit

that could be derived from the project and its results.

For all this,

I GIVE MY CONSENT TO: ________________________________________ to participate in the project: ***Effectiveness in promoting and prescribing exercise in patients with type 2 diabetes: a multicomponent intervention based on the model of basic social processes through Nordic Walking***

That the team of researchers from the University of Vic - Central University of Catalonia and

the main researcher Guillem Jabardo Camprubí can manage my personal data and disseminate the information generated by the project. They are guaranteed to be preserved at all times my identity and privacy, with the guarantees established by Organic Law 3/2018, of 5 of December, Protection of Personal Data and guarantee of digital rights and the Regulation general (EU) 2016/679 of April 27, 2016 on data protection and regulations complementary

That the team of investigators led by Guillem Jabardo Camprubí will keep all the records

carried out on my person in a digital-electronic medium, with the guarantees and the

legally provided deadlines, if they are established, and in the absence of legal provision, during the time that was necessary to fulfill the functions of the project for which the data was obtained.

To __________________________to ____ of______________ of______

Participant Signature: IP Signature:
